# Use of frailty assessment instruments in nephrology populations: a scoping review

**DOI:** 10.1186/s12877-023-04101-y

**Published:** 2023-07-21

**Authors:** Alice L. Kennard, Suzanne Rainsford, Nicholas J. Glasgow, Girish S. Talaulikar

**Affiliations:** 1 Department of Renal Medicine, The Canberra Hospital, Canberra Health Services, Building 15, Yamba Drive, Garran, ACT 2605 Australia; 2 Australian National University, Canberra, ACT Australia

**Keywords:** Frailty, Assessment tools, Scoping review, Chronic kidney disease, Dialysis, Kidney transplant

## Abstract

**Background:**

Frailty is a clinical syndrome of accelerated aging associated with adverse outcomes. Frailty is prevalent among patients with chronic kidney disease but is infrequently assessed in clinical settings, due to lack of consensus regarding frailty definitions and diagnostic tools. This study aimed to review the practice of frailty assessment in nephrology populations and evaluate the context and timing of frailty assessment.

**Methods:**

The search included published reports of frailty assessment in patients with chronic kidney disease, undergoing dialysis or in receipt of a kidney transplant, published between January 2000 and November 2021. Medline, CINAHL, Embase, PsychINFO, PubMed and Cochrane Library databases were examined. A total of 164 articles were included for review.

**Results:**

We found that studies were most frequently set within developed nations. Overall, 161 studies were frailty assessments conducted as part of an observational study design, and 3 within an interventional study. Studies favoured assessment of participants with chronic kidney disease (CKD) and transplant candidates. A total of 40 different frailty metrics were used. The most frequently utilised tool was the Fried frailty phenotype. Frailty prevalence varied across populations and research settings from 2.8% among participants with CKD to 82% among patients undergoing haemodialysis. Studies of frailty in conservatively managed populations were infrequent (*N* = 4). We verified that frailty predicts higher rates of adverse patient outcomes. There is sufficient literature to justify future meta-analyses.

**Conclusions:**

There is increasing recognition of frailty in nephrology populations and the value of assessment in informing prognostication and decision-making during transitions in care. The Fried frailty phenotype is the most frequently utilised assessment, reflecting the feasibility of incorporating objective measures of frailty and vulnerability into nephrology clinical assessment. Further research examining frailty in low and middle income countries as well as first nations people is required. Future work should focus on interventional strategies exploring frailty rehabilitation.

**Supplementary Information:**

The online version contains supplementary material available at 10.1186/s12877-023-04101-y.

## Background

Frailty is a multisystem clinical syndrome resulting from the accumulation of vascular, inflammatory and age-related insults leading to accelerated aging, increased vulnerability to adverse outcomes and lack of functional reserve over time. There is growing interest in understanding the association between frailty and chronic kidney disease (CKD), a population increasingly characterised by advanced age and case complexity [1, 2].

Frailty is common among patients with kidney disease and becomes more prevalent as kidney disease progresses, even after adjustment for age and comorbidity [2, 3]. Frailty is related, but a separate construct, to chronological age, emerging in adults with organ failure at an earlier stage to the general population [4, 5]. For example, in an early study of patients undergoing haemodialysis (HD), 73% of the entire study cohort and 64% of those younger than 40 years of age exhibited frailty [6].

An emerging body of literature describes the clinical implications of frailty in nephrology populations including increased risk of hospitalisation and emergency department presentation, institutionalisation and death [7–10]. The presence of frailty out-performs conventional nephrology metrics in predicting kidney disease progression, renal replacement therapy choice, disease complications and patient-level outcomes [7, 11–14]. Routine use of frailty assessment to inform individualised management decisions has been endorsed as best practice by professional bodies but a consensus diagnostic approach remains to be resolved noting that an operational definition of frailty should be multi-dimensional [15–17].

While the medical syndrome of frailty is widely recognised, debate remains over how best to measure frailty in clinical and research settings. Many operational definitions have been introduced to distinguish frailty from “healthy aging”. These models differ in their conceptual foundations, clinical feasibility, frailty domains and their ability to characterise frailty as either a dichotomous or continuous variable. Other definitions distinguish physical frailty from social and cognitive frailty [18, 19]. To facilitate use in clinical settings, frailty assessments should be multi-dimensional, exclusive of the separate construct of disability, sensitive to dynamic changes in frailty status, predictive of relevant outcomes and feasible in resource-limited settings. There are further considerations unique to assessing patients with kidney disease. Within nephrology populations, definitions that emphasise weight loss such as the Fried phenotype [20] risk confounding by fluctuations in fluid status, while classification that incorporate fatigue may over-report frailty if assessed during the immediate post-dialysis period. The Frailty Index [21] is likewise subject to variations in performance relative to dialysis treatment, while the Clinical Frailty Scale [22] relies heavily on subjective clinical impression. Studies consistently demonstrate that self-reported measures over-estimate frailty in patients with kidney disease compared to objective criteria [23, 24], while nephrologist’s subjective assessment of frailty risks misclassification and discrimination, particularly for older patients and females [25]. Although objective assessment of frailty via performance-based measures such as Fried and the Short Physical Performance Battery form the foundation of comprehensive geriatric assessment, evaluations that can be extracted from the electronic medical record offer many advantages including reduced resource requirements and the ability to examine frailty retrospectively. Finally, performance-based tests of frailty may have limited utility in acute health-care settings when cardiovascular compromise or critical illness compel the use of questionnaire-based instruments.

Scoping reviews examining frailty assessment in acute care settings reveal inconsistent use of frailty tools with 89 different instruments applied [26]. To our knowledge, no scoping reviews have focussed on frailty measures in the CKD, dialysis and kidney transplant context. We argue that frailty reflects vulnerability and is not isolated to a specific modality of renal replacement therapy, and that because individual patients transition between different renal replacement therapy modalities and there is merit in comparing frailty assessment across CKD states. The aim of our scoping review was to examine and clarify key frailty concepts in the context of kidney disease, evaluate the most commonly utilised frailty assessment tools in nephrology research settings, describe the methodological contexts of frailty assessment and identify gaps in knowledge to guide future research work. Our focus is on the construct of physical frailty, acknowledging the existing scoping work exploring cognitive frailty in this patient population [27].

## Methods

This scoping review is reported according to the Preferred Reporting Items for Systematic Reviews and Meta-Analyses extension for Scoping Reviews (PRISMA-ScR) [28].

### Selection criteria and search strategy

The search strategy was developed by AK and SR, with the assistance of a research librarian. This scoping review included original research articles published since January 2000 until November 30 2021. This timeframe was selected to reflect the emergence of the Frailty Phenotype and Frailty Index, developed in 2001, and following which the body of literature examining frailty expanded [20, 21]. This also coincides with the introduction of estimated Glomerular Filtration Rate which allowed for standardisation of CKD definitions [29]. The following inclusion criteria were applied to study selection: participants aged 18 years or older, diagnosed with chronic kidney disease, end stage kidney disease (ESKD) undergoing dialysis or managed conservatively, or undergoing evaluation for, or receipt of, a kidney transplant. Included studies were published in English language in a peer-reviewed journal. Exclusion criteria specified studies based on case reports or qualitative data, study populations with acute kidney injury and intensive care unit setting. As existing work has examined screening for cognitive impairment in dialysis populations [27], we also excluded papers examining cognitive frailty as a distinct construct. No specific patient outcomes were of interest as we sought to identify all adverse outcomes that the included studies reported on.

We searched Medline, CINAHL, Embase, PsychINFO, PubMed and Cochrane Library. Additional articles were identified by searching the reference lists of systematic and literature reviews focusing on frailty in nephrology populations. Grey literature sources were not included. A search strategy was developed to identify frailty instruments, the population evaluated and context for the assessment (see Search Strategy in Supplemental Material). The search was performed on 30^th^ November 2021. Backwards searching and hand searching (searching key references including systematic reviews for other relevant publications, as well as researcher-initiated database searches) were performed to interrogate the reliability of the search strategy. Identified papers were catalogued in EndNote, where duplicates were excluded, then imported to Colandr software (www.Colandrapp.com). Two members of the review team independently screened titles and abstracts that met the inclusion criteria. Disagreements were resolved through discussion with reference to the inclusion criteria, seeking consensus in line with PRISMA-ScR Guidelines. Full-text review and data extraction was performed by all members of the reviewing team reviewers, with source data verification performed for 20% of the included articles to ensure reliability and replicability. The following data was extracted from each included article: year and country of publication, study design, study setting, number of participants, median duration of follow-up, nephrology population enrolled, dialysis vintage, participant age and sex, frailty measurement tool used, context of frailty assessment (when assessed and who completed evaluation), reason for measuring frailty, reported frailty scores, prevalence of frailty, outcome measures examined in relation to frailty.

Descriptive statistical analyses and graphics were conducted using Stata SE 17.0.

## Results

**Fig. 1:**
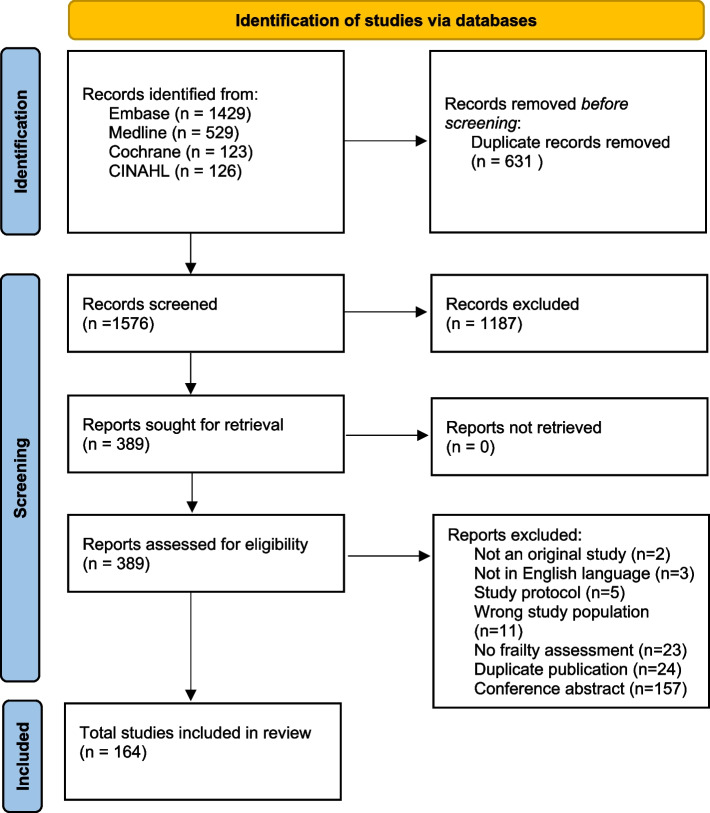
PRISMA 2020 flow diagram for new systematic reviews which included searches of databases and registers only

We found 1576 unique records using the search strategy. Full text was obtained for 389 articles. There were 164 studies comprising 116,005 participants meeting the inclusion criteria for final analysis. See Fig. 1.

### Study characteristics

**Fig. 2:**
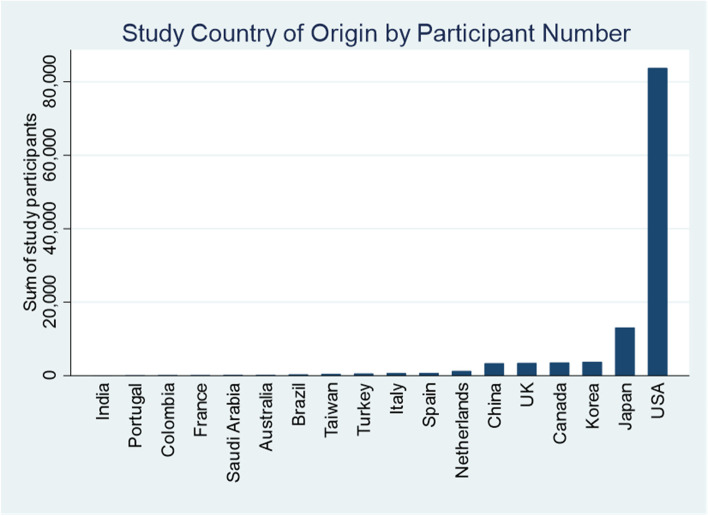
Number of study participants according to country of study origin

The characteristics of included studies are presented in Table 1. The included studies were published between 2004 and 2021, with the majority of studies published in the last 5 years (79%). The included studies were conducted across 18 countries, and 57 out of 164 studies, comprising 30,594 participants were conducted in the USA. Organisation for Economic Cooperation and Development (OECD) high income countries were also over-represented. See Fig. 2.

**Table 1:** Characteristics of the included studies

Nephrology Population	Chronic kidney disease	Haemodialysis	Peritoneal Dialysis	Conservative management	Transplant candidate	Transplant recipient	Total studies (number of participants)
**Number of studies (number of participants)**	39 (41,104)	92 (26,332)	28 (5545)	4 (160)	19 (35,308)	15 (7556)	164 ^a^ (116,005)
**Year of publication**							
2000–2005	1	-	-	-	-	-	1
2006–2010	1	2	1	-	-	-	3
2011–2015	8	18	3	-	1	4	31
2016-current	29	72	24	4	18	11	129
**Country**							
Australia	2	1	1	1	-	-	3 (211)
Brazil	1	2	-	1	-	1	5 (350)
Canada	3	13	6	-	-	-	17 (3596)
China	1	4	7	-	-	-	12 (3400)
Colombia	-	1	1	-	-	-	2 (148)
France	-	-	-	-	1	-	1 (156)
India	-	1	-	-	-	-	1 (39)
Italy	3	1	1	-	-	-	4 (701)
Japan	3	9	1	-	-	1	14 (13,114)
Korea	1	5	2	-	-	-	6 (3759)
Netherlands	2	6	4	1	2	-	8 (1292)
Portugal	-	1	-	-	-	-	1 (83)
Saudi Arabia	-	1	-	-	-	-	1 (205)
Spain	2	2	-	-	-	-	4 (717)
Taiwan	1	8	-	-	-	-	9 (446)
Turkey	-	1	-	-	-	-	1 (579)
UK	10	12	3	1	-	1	18 (3450)
USA	10	23	2	-	16	12	57 (30,594)
**Study setting**							
Single centre	23	48	17	2	11	10	95
Multicentre	16	44	11	2	8	5	69
**Study design**							
Cross-sectional	15	35	7	2	4	2	54
Prospective	20	46	18	2	13	12	91
RCT	2	1	-	-	-	-	3
Retrospective	2	10	3	-	2	1	16
**Purpose of frailty assessment (%)**							
Outcome measure	24 (61.5)	62 (67.4)	20 (71.4)	4 (100)	12 (63.1)	8 (53.3)	104 (63.4)
Risk stratification	12 (30.8)	29 (31.5)	8 28.5)	-	6 (31.6)	7 (46.7)	56 (34.1)
Inclusion/exclusion criterion	2 (5.1)	1 (1.1)	-	-	1 (5.3)	-	4 (2.4)
**Mean age of participants (SD)**							
# of reporting articles	22	72	20	1	12	10	116
Combined mean (SD)	75.6 (11.4)	61.3 (14.9)	59.9 (14.9)	78 (7.0)	54.3 (13.3)	53.6 (14.1)	62.3 (15.9)
**Percentage of males** **Median (IQR)**	57 (50–63)	59 (53.4–63)	60 (55.2–66)	47.6 (45.7–64)	61.1 (60–63.1)	62 (60.2–62.7)	59.4 (53.6–63)
**Mean dialysis duration (SD) (months)**							
# of reporting articles		16 initiation 19 prevalent	12 initiation 4 prevalent				28 initiation 23 prevalent
Combined mean (SD)		68.1 (69.1)	52.9 (51.3)				62.7 (63.8)

^a^ 28 studies included more than one nephrology population

The majority of the studies were prospective observational studies (91 studies), with 54 cross-sectional studies, 16 retrospective analyses and 3 randomised controlled trials. The focus of most of studies was to describe frailty as an outcome measure (104 studies). Frailty assessment was used for risk stratification purposes in 56 studies and formed study inclusion/exclusion criteria in 4 studies. There were 95 single-centre studies and 69 multicentre studies. The majority of studies enrolled more than 100 participants (119 studies).

**Fig. 3:**
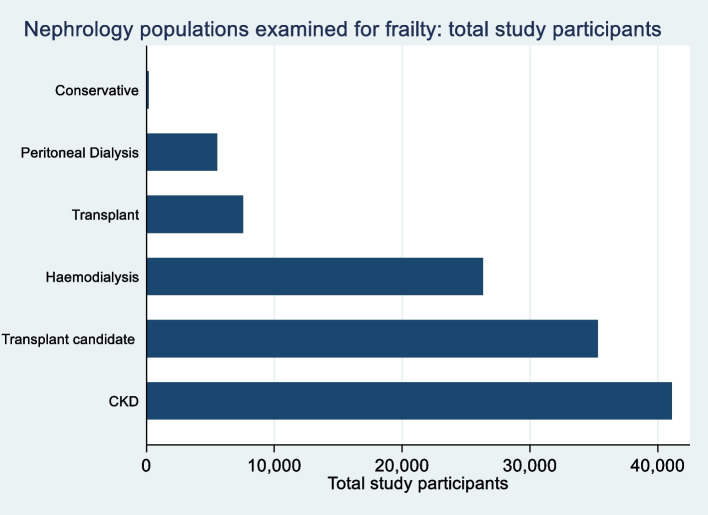
Nephrology populations examined for frailty

Thirty-nine studies comprising 41,104 participants examined frailty in CKD populations [2, 3, 30–65]. Ninety-two studies comprising 26,332 participants enrolled HD populations [5, 6, 9, 10, 25, 30–33, 41, 66–146], 28 studies comprising 5545 participants examined peritoneal dialysis (PD) populations [6, 66–71, 73–83, 147–155] and 19 (35,308 participants) and 14 studies (7556 participants) were conducted in patients undergoing transplant assessment [5, 13, 156–172] or in receipt of a kidney transplant [5, 72, 156, 157, 173–182], respectively. Just four studies of 160 participants examined frailty in conservatively managed ESKD populations [71, 74, 77, 183]. See Fig. 3. Twenty-eight studies enrolled more than one nephrology population.

Mean age of participants was 62.3 ± 15.9 years, with older participants prevalent among CKD studies (75.6 ± 11.4 years) and conservatively managed populations (78.0 ± 7.0 years). Transplant candidates and transplant recipients were, on average, younger (54.3 ± 13.3 years and 53.5 ± 14.1 years, respectively). Males outnumbered females in all studies except for conservatively managed populations.

### Frailty assessment

Frailty assessment characteristics of the included studies are presenting in Table 2. Context of frailty assessment was, in general, poorly described in studies with 61% of studies failing to describe timing of frailty assessment and 64.6% of studies not reporting who performed the frailty assessment. Where included studies provided this detail, 16 studies (41%) examined frailty in participants with CKD in outpatient settings while transplant candidates where most frequently assessed at admission for transplantation operation (8 studies, 53.3% of studies). Assessments for frailty were most commonly performed by researchers rather than clinical staff (51 studies, 31%).

**Table 2:** Frailty assessment tools

Nephrology Population	Chronic kidney disease	Haemodialysis	Peritoneal Dialysis	Conservative management	Transplant candidate	Transplant recipient	Total
References	2, 3, 30–65	5, 6, 9, 10, 25, 30–33, 41, 66–146	6, 66–71, 73–83, 147–155	71, 74, 77, 183	5, 13, 156–172	5, 72, 156, 157, 173–182	
**Number of studies**	39	92	28	4	19	15	164^a^
**When frailty was assessed n(%)**							
Chart review	2 (5.1)	2 (2.2)	2 (7.1)	-	-	1 (6.7)	7 (4.3)
Outpatient setting	16 (41)		2 (7.1)	1 (25)	6 (31.6)	1 (6.7)	26 (15.9)
At admission	-	1 (1.1)	-	-	4 (21.1)	8 (53.3)	13 (7.9)
Non-dialysis day		4 (4.4)			-	-	4 (2.4)
Before dialysis		10 (10.9)			-	-	10 (6.1)
On dialysis		6 (6.5)			-	-	6 (3.7)
Mixed	2 (5.1)	9 (9.8)	6 (21.4)	1 (25)	5 (26.3)	3 (20)	26 (15.9)
Not reported	19 (48.7)	55 (59.8)	18 (64.3)	2 (50)	4 (21.1)	2 (13.3)	100 (61.0)
**Who performed frailty assessment**							
Patient self-report	4 (10.2)	16 (17.4)	4 (14.3)	-	-	1 (6.7)	25 (15.2)
Researcher	8 (20.5)	22 (23.9)	11 (38.3)	3 (75)	4 (21.1)	3 (20)	51 (31.1)
Doctor	3 (7.7)	7 (7.6)	5 (17.9)	-	1 (5.3)	-	16 (9.8)
Nurse	3 (7.7)	6 (6.5)	3 (10.7)	-	3 (15.8)	-	15 (9.1)
Exercise physiologist or physiotherapist	1 (2.7)	1 (1.1)		-		-	2 (1.2)
Caregiver	1 (2.7)	1 (1.1)	1 (3.6)	-	-	-	3 (1.8)
Not reported	23 (58.9)	50 (54.3)	10 (35.7)	1 (25)	11 (57.9)	11 (73.3)	106 (64.6)
**Frailty tool used n(%)**							
Fried phenotype	21 (53.8)	50 (54.3)	9 (32.1)	2 (50)	13 (68.4)	12 (80)	90 (54.9)
Clinical frailty scale	10 (25.6)	18 (19.6)	11 (39.2)	1 (25)	-	1 (6.7)	29 (17.7)
Frailty Index	7 (17.9)	5 (5.4)	2 (7.1)	-	-		9 (5.5)
Short physical battery	5 (12.8)	3 (3.2)	1 (3.6)	-	2 (10.5)	1 (6.7)	10 (6.1)
ADLs and IADLs	5 (12.8)	9 (9.8)	4 (14.3)	1 (25)	-	-	15 (9.1)
Comprehensive geriatric assessment	1 (2.6)	7 (7.6)	3 (10.7)	1 (25)	1 (5.3)	-	8 (4.9)
FRAIL scale	1 (2.6)	12 (13.0)	2 (7.1)	-	-	-	13 (7.9)
In-house questionnaire	-	-	6 (21.4)	-	-	-	6 (3.7)

^a^ 28 studies included more than one nephrology population

A total of 40 different frailty metrics were used across 164 studies. Most studies included only one frailty measure (*n* = 107), while 27 studies employed 2 different frailty metrics. Thirty studies included 3 or more frailty measures. The most frequently utilised tool for frailty assessment was Fried frailty phenotype with 90 studies (54.9%) utilising this metric (Table 2). Clinical Frailty Scale was also frequently used (29 studies), particularly among PD populations. Six studies reported subjective clinical assessments by doctors and 4 studies reported nursing staff clinical impression. Patient perception was sought in one study and 1 study reported caregiver perception. An in-house questionnaire of Chinese PD populations [153] was used in 6 Chinese PD studies. Comprehensive geriatric assessment was infrequently used across study populations (4.9% of studies).

**Fig. 4:**
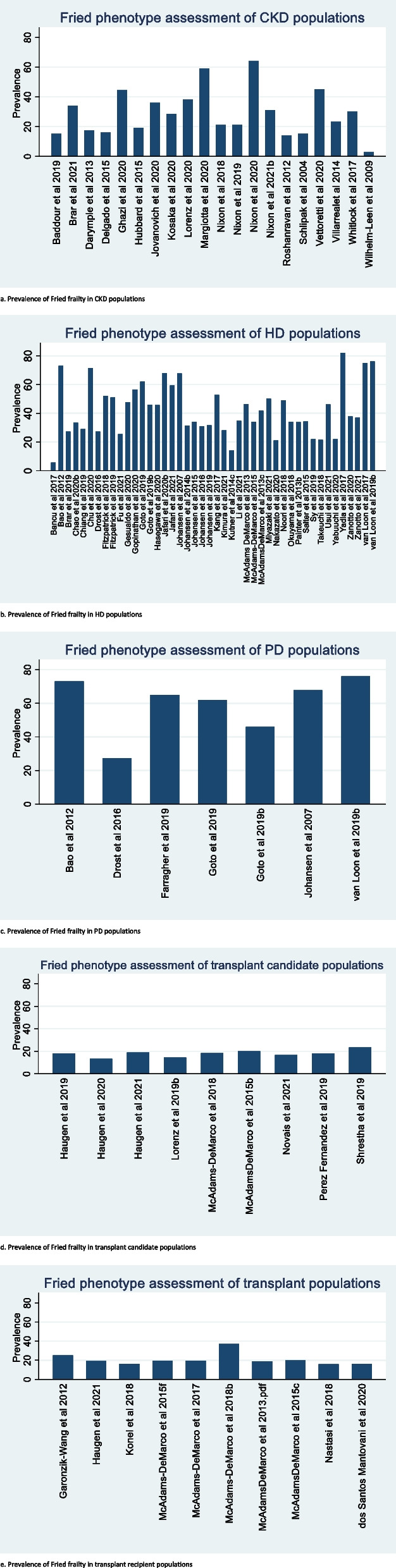
**a**. Prevalence of Fried frailty in CKD populations. **b**. Prevalence of Fried frailty in HD populations. **c**. Prevalence of Fried frailty in PD populations. **d**. Prevalence of Fried frailty in transplant candidate populations. **e**. Prevalence of Fried frailty in transplant recipient populations

One hundred and forty-five studies reported frailty prevalence rate. Fried frailty prevalence varied across studies and study populations, with highest prevalence of Fried-phenotype frailty reported in HD (5.6% to 82%) and PD (27.3% to 76%) populations and lower Fried-phenotype frailty evident in studies evaluating participants with CKD (2.8% to 44.4%), transplant candidates (13.3% to 23.4%) and transplant recipients (15.7% to 37%) (see Fig. 4a-e). Study population demographics are presented in Supplemental Tables 4, 5, 6, 7 and 8, demonstrating the heterogenous study population characteristics, particularly in studies focussing on CKD and HD populations. Only one study utilising Fried phenotype reported prevalence within conservatively managed populations, describing a prevalence rate of 62% [71]. Use of Fried phenotype in six studies facilitated description of an intermediately or pre-frail frail patient population, reporting a prevalence rate of 28.4–37.7% [9, 122, 162, 179, 180].

Prevalence of frailty based on other assessment tools is presented in Supplemental Materials, stratified by study population. We present a comparison of these reported prevalence rates where a consistent tool is used in three or more published studies. Frailty prevalence varies both by study population and frailty metric.

Six studies performed frailty assessment at more than one time point, demonstrating frailty progression and increase in frailty prevalence within the CKD and PD study cohorts followed for mean 45.7 ± 6.0 months [149], at 12 and 24 months of follow-up median 4 years [59] and unspecified [184]. One study of HD patients assessed at baseline, 12 months and 24 months follow-up demonstrated improvement in frailty parameters as often as worsening [109]. Among transplant patients, serial frailty assessment demonstrated improvement [178] or varied responses [159] following kidney transplantation.

### Frailty outcomes

Frailty was found to be predictive of patient outcomes in 133 of 143 studies that evaluated clinical sequelae. Forty studies reported that frailty predicted mortality outcomes [6, 9, 10, 30, 37, 39, 46, 52, 61, 65, 66, 69, 72, 75, 83, 84, 92, 95, 97, 100, 104, 109, 110, 124, 145, 147, 152–155, 157, 159, 162, 163, 166, 175, 180, 182, 185, 186]  and 16 studies found frailty was associated with hospitalisation [6, 9, 10, 30, 41, 46, 66, 69, 81, 83, 84, 116, 138, 153, 154, 177]. Three studies reported frailty predicted increased likelihood of in-centre HD modality choice compared to a home-based dialysis modality [39, 61, 187]. Eight studies reported frailty was associated with reduced likelihood of transplant referral and waitlisting, removal from waitlist or death on waitlist [66, 75, 150, 158, 160, 161, 165, 188] while four studies reported an association with post-transplant complications [168, 173, 175, 179].

Four studies compared and reported on the sensitivity and specificity of different frailty metrics, comparing them to a gold standard, alternatively defined as comprehensive geriatric assessment [136], Frailty Index [33, 76] and Fried phenotype [58]. These studies report the Clinical Frailty Scale [76] and Fried phenotype are highly specific [136], but with corresponding lower sensitivity, and that the Groningen Frailty index has comparable discriminatory ability [33]. Subjective frailty assessments based on self-rated health and a Surprise Question have a high negative predict value, useful for excluding frailty [58].

## Discussion

This scoping review synthesises the published literature concerned with frailty assessment in a number of diverse nephrology populations. We found this to be an emerging field of research with most studies conducted in the last 6 years. Published literature to date has extensively examined CKD populations, dialysis and transplant populations but few studies have examined conservatively managed patients with end stage kidney disease. This differs markedly from clinical practice where conservatively managed patients seeking renal supportive care or palliative care are a major focus of frailty assessment [189]. Patients engaged in PD are also underexamined by the current published literature exploring frailty, as has been reported elsewhere [190]. Frailty occurs with high but variable prevalence among CKD and dialysis populations, revealing study population heterogeneity and utilisation of different frailty metrics with varying sensitivities. Frailty is less prevalent among patients undergoing evaluation for transplant candidacy and transplant recipients, likely reflecting clinicians’ awareness of the implications of frailty in transplant outcomes and the health economics informing transplant allocation. There is robust evidence that frailty is a risk factor for adverse clinical outcomes, suggesting that frailty assessment may improve risk stratification and advance communication in clinical interactions. The focus of studies performed to date has been descriptive and prognostic, with little research activity exploring interventions for frailty rehabilitation.

Study settings reveal an over-representation of middle- and higher-income countries where frailty is likely to be less prevalent. There is little exploration of frailty in first-nations people, reinforcing concerns that frailty phenotype in indigenous populations may be under-recognised and contribute to inequities in health care delivery [191, 192]. Study populations demonstrate under-representation of female patients, despite recognition that female gender increases odds of frailty [81].

Frailty may be assessed either by subjective (self-reported or clinician perception) or objective means, using direct measurement of physical performance or exploiting descriptive tools with clear categorical definitions. Overall, there were 40 different frailty measures used across 164 studies. The range of instruments utilised by studies included in this review reflects the lack of consensus regarding the best instruments for assessing frailty [193, 194]. This heterogeneity has similarly been reported by scoping reviews examining frailty assessment in acute care settings [26] and solid organ transplantation [195]. Assessment settings and contexts were poorly described across studies, although the majority of studies that did provide this detail indicated a propensity for CKD patient assessment in the outpatient setting, while transplant candidates were most commonly assessed at admission for kidney transplantation surgery. The utilisation of frailty assessments in these settings suggests capacity and feasibility to incorporate frailty evaluation into routine fast-paced high-turnover nephrology assessment.

A small number of studies evaluated frailty at more than one time point, demonstrating, in general, a progression and increase in frailty prevalence among CKD and dialysis cohorts. In contrast, those studies exploring frailty dynamics in transplant populations reported improvement in frailty parameters or mixed but nonetheless changes in frailty state post transplantation. There were no studies reporting on frailty progression or rehabilitation over the course of an acute hospital admission. Assessment from frailty in the hospital setting has a number of challenges due to the severity of health status of hospitalised older adults, risk of delirium and medication changes as well as pragmatic considerations of the environment [196]. Nonetheless, acute hospital admission is a well-recognised risk factor for frailty progression within the geriatric literature [196–198]. As commentators on this issue point out, it is only with the implementation of frailty assessment as hospital admission that we can prevent the emergence of new cases of frailty and the occurrence of adverse outcomes [199]. It has been reported that frequent hospitalisation among patients undergoing HD is associated with greater frailty at any point and worsening frailty over time [109, 200], but how frailty behaves across an acute admission event among patients with kidney disease remains unknown. Especially relevant for nephrology populations prone to frequent hospitalisation events, studies that examine the frailty syndrome at discharge and following hospitalisation compared to admission are necessary to identify the occurrence of transitions between degrees of frailty (progression and reversion) and understand how frailty can be remedied.

Frailty assessment was predominantly performed by specifically enlisted research staff, with infrequent assessment by clinical or allied health professionals. Where clinicians were involved in the assessment of frailty, this favoured subjective assessment, a measure previously demonstrated to be unreliable with a high risk of bias [25].This suggests a need to build capacity and experience within the nephology workforce in objective frailty clinical assessment. The patient and caregiver perspectives of frailty remain under-explored.

In this review, the most frequently used instrument for assessing frailty was the Fried frailty phenotype which combines self-reported components (fatigue/exhaustion, low physical activity) alongside assessments of physical function (grip strength, walk speed) and biometrics (unintentional weight loss). The Clinical Frailty Scale lends itself well to retrospective analyses and was favoured by studies utilising this methodology. Frailty Index was frequently utilised in CKD studies where competing comorbidities were of greater or equivalent relevance. Of note, frailty metrics borrowed from palliative care such as the Karnovsky and Surprise Question were infrequently used in research settings. Chinese PD studies favoured the use of the in-house questionnaire but its utility outside of this population demographic remains untested. Frailty assessments among participants with solid organ transplant populations favour the Fried frailty phenotype [195] while geriatric publications have preferred the Frailty Index [199]. Assessments in acute care settings have equally relied on the Fried frailty phenotype, Frailty Index and Clinical Frailty Scale [26]. It is likely that different instruments offer distinctive advantages in varied clinical settings.

We were able to compare rates of Fried frailty reported in different study settings. This analysis demonstrates highly variable rates of frailty, particularly among CKD and HD populations, likely reflecting heterogenous population characteristics as well as differences in health care access and policy.

Use of the Fried phenotype afforded description of intermediately frail or pre-frail patients in six studies, suggesting greater flexibility for this frailty assessment tool. The Frailty Index, FRAIL scale, Cardiovascular Health Study Index and Study of Osteoporotic Fractures Index also have the capacity to define intermediate states off frailty but was rarely utilised by included studies. The rates of pre-frailty reported among nephrology populations corresponds with similar prevalence of pre-frailty reported in the geriatric literature [201–210]. Importantly, pre-frailty is a dynamic state with the potential for reversion to the state of robustness [20, 211], suggesting the opportunity for early intervention to improve or maintain health status and prevent functional decline.

We verified that frailty predicts higher risk of adverse patient outcomes including mortality, hospitalisation, restricted renal replacement therapy choice, reduced likelihood of transplantation and post-transplantation complications. Earlier systematic review reported similar adverse outcomes [212]. We propose that there is now sufficient literature exploring kidney disease and frailty outcomes to justify future meta-analysis.

Whether early identification of frailty will allow intervention and treatment remains to be seen. Frailty management guidelines focus on community dwelling older adults [213, 214]. The importance of frailty is also recognised by the discipline of cardiology where an “Essential frailty toolkit” and consensus documents specifying strategies for primary, secondary and tertiary frailty prevention guide management [215–217]. This review highlights a critical lack of interventional studies exploring frailty management strategies.

The broad scope of this review emerges as a strength, allowing clinicians and researchers to consider the evidence relevant to their individual patient and their position with CKD states. This scoping review was restricted to studies published in English, potentially leading to underrepresentation of the non-English speaking population and the value of non-English frailty instruments. We sought to apply quality controls by including only papers which had undergone robust peer review processes. Through exclusion of conference abstracts, we may have missed contemporary research, thereby limiting the applicability of our conclusions. By design, scoping reviews do not contain a quality of evidence assessment and this review subsequently provides a descriptive study of available research, including gaps in evidence. Furthermore, our review excluded studies set within ICU and performed in patients with acute kidney injury; our findings do not apply to these patient populations.

## Conclusions

This scoping review describes a rapidly proliferating body of literature concerned with frailty in nephrology populations. We verify a high prevalence of frailty among heterogeneous patient populations and the utilisation of a variety of assessment tools in diverse research settings. The Fried frailty phenotype is the most commonly used frailty metric, characterised by a high specificity and facilitating the valuable identification of a vulnerable pre-frail state. There is robust evidence that frailty predicts adverse patient outcomes and may augment traditional risk-stratification and decision-making tools in nephrology clinical practice. There is a need for further studies examining frailty in culturally and linguistically diverse populations and an urgent need for interventional research exploring frailty rehabilitation strategies.

## Supplementary Information

**Additional file 1.** Search Strategy.

**Additional file 2: Supplemental Figure 1.** a. Prevalence of frailty based on assessment by Frailty Index in CKD populations. b. Prevalence of frailty based on assessment by Clinical Frailty Scale in CKD populations. c. Prevalence of frailty based on assessment by Clinical Frailty Scale in HD populations. d. Prevalence of frailty based on assessment by FRAIL scale in HD populations. e. Prevalence of frailty based on assessment by Frailty Index of HD populations. f. Prevalence of frailty based on assessment by Clinical Frailty Scale of PD populations.

**Additional file 3: Supplementary Table 4.** Studies examining patient populations with chronic kidney disease using Fried assessment of frailty.** Supplementary Table 5.** Studies examining patient populations undergoing haemodialysis using Fried assessment of frailty.** Supplementary Table 6.** Studies examining patient populations undergoing peritoneal dialysis using Fried assessment of frailty.** Supplementary Table 7.** Studies examining patient populations assessed for kidney transplant candidacy using Fried assessment of frailty.** Supplementary Table 8.** Studies examining patient populations with kidney transplant using Fried assessment of frailty.

## Data Availability

As a scoping review, raw data are available from the individual publications included in the reference list. Further detail is available from the corresponding author on reasonable request.

## Abbreviations

CKD: Chronic kidney disease
Conservative: Conservatively managed kidney disease
ESKD: End stage kidney disease
HD: Haemodialysis
PD: Peritoneal dialysis
OECD: Organisation for Economic Cooperation and Development
