# Supplementary material for: Use of frailty assessment instruments in nephrology populations: a scoping review
Source: BMC Geriatr. 2023 Jul 21;23:449. doi: 10.1186/s12877-023-04101-y (PMC10360289; doi:10.1186/s12877-023-04101-y)
Supplement: Supplementary file 1 — Additional file 1. Search Strategy. [file 12877_2023_4101_MOESM1_ESM.docx]

Supplemental Material: Search Strategy

Embase

| Search number | Search term | Results |
| --- | --- | --- |
| 1 | *kidney failure/ | 34608 |
| 2 | *renal replacement therapy/ | 18500 |
| 3 | exp *hemodialysis/ | 49753 |
| 4 | *peritoneal dialysis/ | 17918 |
| 5 | *kidney transplantation/ | 74845 |
| 6 | renal insufficiency.ti,ab. | 28594 |
| 7 | Chronic kidney failure.ti,ab. | 1648 |
| 8 | H?emodialysis.ti,ab. | 112676 |
| 9 | CKD.ti,ab. | 65417 |
| 10 | ((kidney* or renal) adj (failure* or disease* or insufficien* or disorder*)).ti,ab. | 353482 |
| 11 | ((kidney* or renal) adj replacement therap*).ti,ab. | 26477 |
| 12 | ((renal or kidney*) adj (transplant* or graft* or replac* or artificial* or allograft* or dialys*)).ti,ab. | 162100 |
| 13 | *frail elderly/ | 4990 |
| 14 | *geriatric assessment/ | 6302 |
| 15 | *geriatric patient/ | 10672 |
| 16 | frail*.mp. | 48385 |
| 17 | exp functional status assessment/ | 182876 |
| 18 | Edmonton Frail Scale/ | 254 |
| 19 | "Functional Assessment of Chronic Illness Therapy Fatigue Scale"/ | 755 |
| 20 | Edmonton frail scale*.mp. | 374 |
| 21 | ((Timed Up and Go Test) or TUG).mp. | 10285 |
| 22 | (SF-36 tool* or SF-36 instrument* or SF-36 score* or SF-36 scale* or SF-36 assess*).mp. | 5906 |
| 23 | "Kidney Disease Quality of Life Instrument".mp. | 55 |
| 24 | ((FRAIL or Fried frailty or fried frailty phenotype) adj2 (scale* or score* or index* or assess*)).mp. | 1542 |
| 25 | (Fried frailty or fried frailty phenotype or fried phenotype).mp. | 902 |
| 26 | (Karnovsky adj2 (assess* or scale)).mp. | 23 |
| 27 | ((grasp* or grip* strength*) adj2 (test* or score* or scale* or instrument* or tool* or assess*)).ti,ab. | 3492 |
| 28 | ((validat* or screen* or scale* or scor* or predic*) adj4 (tool* or metric* or measure* or index* or assess*)).ti,ab. | 594428 |
| 29 | preval*.ti,ab. | 1194022 |
| 30 | predic*.ti,ab. | 2376310 |
| 31 | or/1-12 | 581645 |
| 32 | or/13-16 | 63011 |
| 33 | or/17-30 | 3906040 |
| 34 | 31 and 32 and 33 | 1429 |

Medline:

| Search number | Search term | Results |
| --- | --- | --- |
| 1 | *Renal Insufficiency/ | 10844 |
| 2 | *renal replacement therapy/ | 3631 |
| 3 | exp *Renal Dialysis/ | 86137 |
| 4 | *Peritoneal Dialysis/ | 14257 |
| 5 | *kidney transplantation/ | 83135 |
| 6 | renal insufficiency.ti,ab. | 21270 |
| 7 | Chronic kidney failure.ti,ab. | 1220 |
| 8 | H?emodialysis.ti,ab. | 72906 |
| 9 | CKD.ti,ab. | 28464 |
| 10 | ((kidney* or renal) adj (failure* or disease* or insufficien* or disorder*)).ti,ab. | 218573 |
| 11 | ((kidney* or renal) adj replacement therap*).ti,ab. | 12769 |
| 12 | ((renal or kidney*) adj (transplant* or graft* or replac* or artificial* or allograft* or dialys*)).ti,ab. | 97120 |
| 13 | *frail elderly/ | 8269 |
| 14 | geriatric patient.ti,ab. | 14112 |
| 15 | *geriatric patient/ | 1432 |
| 16 | frail*.mp. | 28018 |
| 17 | functional status assessment.mp. | 84 |
| 18 | edmonton frail scale.mp. | 118 |
| 19 | "Functional Assessment of Chronic Illness Therapy Fatigue Scale".mp. | 67 |
| 20 | ((Timed Up and Go Test) or TUG).mp. | 4577 |
| 21 | (SF-36 tool* or SF-36 instrument* or SF-36 score* or SF-36 scale* or SF-36 assess*).mp. | 3358 |
| 22 | "Kidney Disease Quality of Life Instrument".mp. | 32 |
| 23 | ((FRAIL or Fried frailty or fried frailty phenotype) adj2 (scale* or score* or index* or assess*)).mp. | 609 |
| 24 | (Fried frailty or fried frailty phenotype or fried phenotype).mp. | 385 |
| 25 | (Karnovsky adj2 (assess* or scale)).mp. | 7 |
| 26 | ((grasp* or grip* strength*) adj2 (test* or score* or scale* or instrument* or tool* or assess*)).ti,ab. | 1937 |
| 27 | ((validat* or screen* or scale* or scor* or predic*) adj4 (tool* or metric* or measure* or index* or assess*)).ti,ab. | 334569 |
| 28 | preval*.ti,ab. | 728796 |
| 29 | predic*.ti,ab. | 1472287 |
| 30 | or/1-12 | 384574 |
| 31 | or/13-16 | 40675 |
| 32 | or/17-29 | 2325718 |
| 34 | 30 and 31 and 32 | 529 |

Cochrane

| Search term | Results |
| --- | --- |
| MeSH descriptor: [Renal Insufficiency] this term only | 1604 |
| MeSH descriptor: [renal replacement therapy] this term only | 195 |
| MeSH descriptor: [Renal Dialysis] explode all trees | 5328 |
| MeSH descriptor: [Peritoneal Dialysis] this term only | 905 |
| MeSH descriptor: [kidney transplantation] this term only | 3648 |
| renal insufficiency:ti,ab | 2369 |
| Chronic kidney failure:ti,ab | 4562 |
| H?emodialysis:ti,ab | 11955 |
| CKD:ti,ab | 6144 |
| ((kidney* or renal) NEAR (failure* or disease* or insufficien* or disorder*)):ti,ab | 28730 |
| ((kidney* or renal) NEAR replacement therap*):ti,ab | 2154 |
| ((renal or kidney*) NEAR (transplant* or graft* or replac* or artificial* or allograft* or dialys*)):ti,ab | 15804 |
| MeSH descriptor: [frail elderly] this term only | 755 |
| geriatric patient:ti,ab | 2410 |
| frail* | 4583 |
| functional status assessment | 9760 |
| edmonton frail scale | 33 |
| "Functional Assessment of Chronic Illness Therapy Fatigue Scale" | 283 |
| ((Timed Up and Go Test) or TUG) | 3905 |
| (SF-36 tool* or SF-36 instrument* or SF-36 score* or SF-36 scale* or SF-36 assess*) | 12978 |
| "Kidney Disease Quality of Life Instrument" | 13 |
| ((FRAIL or Fried frailty or fried frailty phenotype) NEAR/2 (scale* or score* or index* or assess*)) | 867 |
| (Fried frailty or fried frailty phenotype or fried phenotype) | 212 |
| (Karnovsky NEAR/2 (assess* or scale)) | 5 |
| ((grasp* or grip* strength*) NEAR/2 (test* or score* or scale* or instrument* or tool* or assess*)) | 5544 |
| ((validat* or screen* or scale* or scor* or predic*) NEAR/4 (tool* or metric* or measure* or index* or assess*)):ti,ab | 95139 |
| preval*:ti,ab | 45022 |
| predic*:ti,ab | 100493 |
| #1 OR #2 OR #3 OR #4 OR #5 OR #6 OR #7 OR #8 OR #9 OR #10 OR #11 OR #12 | 49742 |
| #13 OR #14 OR #15 | 6687 |
| #16 OR #17 OR #18 OR #19 OR #20 OR #21 OR #22 OR #23 OR #24 OR #25 OR #26 OR #27 OR #28 | 243592 |
| #29 AND #30 AND #31 | 123 |

CINAHL

| Search number | Search term | Results |
| --- | --- | --- |
| 1 | (MM "Renal Insufficiency") | 4218 |
| 2 | (MM "Renal Replacement Therapy") | 1258 |
| 3 | (MM "Dialysis") | 2595 |
| 4 | (MM "Peritoneal Dialysis") | 2518 |
| 5 | (MM "Kidney Transplantation") | 8909 |
| 6 | (MM "Hemodialysis") | 12128 |
| 7 | TI H?emodialysis OR AB H?emodialysis | 3024 |
| 8 | TI CKD OR AB CKD | 10311 |
| 9 | TI ( ((kidney* or renal) N1 (failure* or disease* or insufficien* or disorder*)) ) OR AB ( ((kidney* or renal) N1 (failure* or disease* or insufficien* or disorder*)) | 52875 |
| 10 | TI ( ((kidney* or renal) N2 replacement therap*) ) OR AB ( ((kidney* or renal) N2 replacement therap*) ) | 4535 |
| 11 | TI ( ((renal or kidney*) N1 (transplant* or graft* or replac* or artificial* or allograft* or dialys*)) ) OR AB ( ((renal or kidney*) N1 (transplant* or graft* or replac* or artificial* or allograft* or dialys*)) ) | 17019 |
| 12 | (MM "Frail Elderly") | 5185 |
| 13 | TI geriatric patient OR AB geriatric patient | 3096 |
| 14 | frail | 12835 |
| 15 | functional status assessment | 390 |
| 16 | edmonton frail scale | 92 |
| 17 | "Functional Assessment of Chronic Illness Therapy Fatigue Scale" | 45 |
| 18 | ((Timed Up and Go Test) or TUG) | 3263 |
| 19 | (SF-36 tool* or SF-36 instrument* or SF-36 score* or SF-36 scale* or SF-36 assess*) | 1524 |
| 20 | "Kidney Disease Quality of Life Instrument" | 16 |
| 21 | ((FRAIL or Fried frailty or fried frailty phenotype) N2 (scale* or score* or index* or assess*)) | 576 |
| 22 | (Fried frailty or fried frailty phenotype or fried phenotype) | 307 |
| 23 | (Karnovsky N2 (assess* or scale)) | 2 |
| 24 | TI ( ((grasp* or grip* strength*) N2 (test* or score* or scale* or instrument* or tool* or assess*)) ) OR AB ( ((grasp* or grip* strength*) N2 (test* or score* or scale* or instrument* or tool* or assess*)) ) | 1271 |
| 25 | TI ( ((validat* or screen* or scale* or scor* or predic*) N4 (tool* or metric* or measure* or index* or assess*)) ) OR AB ( ((validat* or screen* or scale* or scor* or predic*) N4 (tool* or metric* or measure* or index* or assess*)) ) | 181009 |
| 26 | TI preval* OR AB preval* | 242197 |
| 27 | TI predic* OR AB predic* | 408529 |
| 28 | S1 OR S2 OR S3 OR S4 OR S5 OR S6 OR S7 OR S8 OR S9 OR S10 OR S11 | 81693 |
| 29 | S12 OR S13 OR S14 | 740827 |
| 30 | S15 OR S16 OR S17 OR S18 OR S19 OR S20 OR S21 OR S22 OR S23 OR S24 OR S25 OR S26 | 15810 |
| 31 | S27 AND S28 AND S29 | 126 |
