# Supplementary material for: Use of frailty assessment instruments in nephrology populations: a scoping review
Source: BMC Geriatr. 2023 Jul 21;23:449. doi: 10.1186/s12877-023-04101-y (PMC10360289; doi:10.1186/s12877-023-04101-y)
Supplement: Supplementary file 2 — Additional file 2: Supplemental Figure 1. a. Prevalence of frailty based on assessment by Frailty Index in CKD populations. b. Prevalence of frailty based on assessment by Clinical Frailty Scale in CKD populations. c. Prevalence of frailty based on assessment by Clinical Frailty Scale in HD populations. d. Prevalence of frailty based on assessment by FRAIL scale in HD populations. e. Prevalence of frailty based on assessment by Frailty Index of HD populations. f. Prevalence of frailty based on assessment by Clinical Frailty Scale of PD populations. [file 12877_2023_4101_MOESM2_ESM.docx]

Supplemental Figure 1a. Prevalence of frailty based on assessment by Frailty Index in CKD populations

Supplemental Figure 1b. Prevalence of frailty based on assessment by Clinical Frailty Scale in CKD populations

Supplemental Figure 1c. Prevalence of frailty based on assessment by Clinical Frailty Scale in HD populations

Supplemental Figure 1d. Prevalence of frailty based on assessment by FRAIL scale in HD populations

Supplemental Figure 1e. Prevalence of frailty based on assessment by Frailty Index of HD populations

Supplemental Figure 1f. Prevalence of frailty based on assessment by Clinical Frailty Scale of PD populations
