# Supplementary material for: Use of frailty assessment instruments in nephrology populations: a scoping review
Source: BMC Geriatr. 2023 Jul 21;23:449. doi: 10.1186/s12877-023-04101-y (PMC10360289; doi:10.1186/s12877-023-04101-y)
Supplement: Supplementary file 3 — Additional file 3: Supplementary Table 4. Studies examining patient populations with chronic kidney disease using Fried assessment of frailty. Supplementary Table 5. Studies examining patient populations undergoing haemodialysis using Fried assessment of frailty. Supplementary Table 6. Studies examining patient populations undergoing peritoneal dialysis using Fried assessment of frailty. Supplementary Table 7. Studies examining patient populations assessed for kidney transplant candidacy using Fried assessment of frailty. Supplementary Table 8. Studies examining patient populations with kidney transplant using Fried assessment of frailty. [file 12877_2023_4101_MOESM3_ESM.docx]

Supplemental Tables

CKD

| Reference name | Reference number | Country | Sample size | Nephrology population | Dialysis vintage | Patient age  Mears Years +/- SD  Median years (range) | Patient gender (% male) |
| --- | --- | --- | --- | --- | --- | --- | --- |
| Baddour_2019 | 58 | USA | 271 | GFR<30 | N/A | 71 (66-77) | 54 |
| Brar_2021 | 60 | Canada | 603 | GFR<30 | N/A | 66 (54-73) | 64 |
| Darymple_2013 | 48 | USA | 4150 | CKD_nonspecific | N/A | 75 | 41 |
| Delgado_2015 | 109 | USA | 812 | CKD_nonspecific | N/A | 42+/-61 | 60.5 |
| Ghazi_2020 | 59 | USA | 1502 | CKD_nonspecific | N/A | 63+/-10 | 56 |
| Hubbard_2015 | 3 | Australia | 110 | CKD_nonspecific | N/A | 65.2+/-14.6 | 53.6 |
| Jovanovich_2020 | 47 | USA | 2376 | GFR>30 | N/A | 73+/-9 | 60 |
| Kosaka_2020 | 50 | Japan | 109 | CKD_nonspecific | N/A | 70.9+/-11.3 | 66.1 |
| Lorenz_2020 | 53 | USA | 21 | GFR<30 | N/A | 62 years (IQR 53-67) | 57.1 |
| Margiotta_2020 | 54 | Spain | 64 | GFR>30 | N/A | 80.7+/-6.2 | 67.2 |
| Nixon_2018 | 32 | UK | 90 | GFR<30; HD | N/A | 69+/-13 | 50 |
| Nixon_2019 | 31 | UK | 90 | GFR<30; HD | N/A | 69+/-13 | 50 |
| Nixon_2020 | 51 | UK | 353 | CKD_nonspecific | N/A |  | 43.5 |
| Nixon_2021 | 30 | UK | 450 | GFR<30; HD | N/A | 80+/-19 | 57 |
| Roshanravan_2012 | 2 | USA | 336 | CKD_nonspecific | N/A | 59+/-13 | 81 |
| Schlipak_2004 | 57 | USA | 5888 | GFR>30 | N/A | Mean 76 | 61 |
| Vettoretti_2020 | 34 | Italy | 112 | GFR>30; GFR<30 | N/A | 80+/-6 | 70 |
| Whitlock_2017 | 61 | Canada | 508 | GFR<30 | N/A | Not reported | 57 |
| Wilhelm-Leen_2009 | 52 | USA | 10256 | CKD_nonspecific | N/A | 49.6+/-1.3 | Not reported |

Supplementary Table 4: Studies examining patient populations with chronic kidney disease using Fried assessment of frailty.

HD

| Reference name | Reference number | Country | Sample size | Nephrology population | Dialysis vintage | Patient age  Mears Years +/- SD  Median years (range) | Patient gender (% male) |
| --- | --- | --- | --- | --- | --- | --- | --- |
| Bancu_2017 | 186 | Spain | 320 | HD | Not reported | 70.26+/-13.85 | 59.4 |
| Bao_2012 | 6 | USA | 1576 | HD; PD | Initiation | 59.6+/-14.2 | 55.5 |
| Brar_2019 | 145 | Canada | 109 | Home dialysis | Not reported | 64 (51-73) | 68 |
| Chao_2020b | 92 | Taiwan | 33 | HD | 45.6+/-33.6 months | 69.5+/-9.4 | 45 |
| Chiang_2019 | 93 | USA | 440 | HD | Not reported | 56.1+/-14.2 | 100 |
| Chu_2020 | 5 | USA | 6078 | HD; transplant-candidate; Transplant | Initiation | 55 (IQR 47+/-63) |  |
| Fitzpatrick_2018 | 95 | USA | 370 | HD | Initiation | 54.9+/-13.1 | 58 |
| Fitzpatrick_2019 | 96 | USA | 285 | HD | 11 months (9.3-15) | 55+/-13 | 57 |
| Fu_2021 | 97 | China | 208 | HD | 82.0 (69.0-142.5) months | 60.5±12.7 | 54.3 |
| Gesualdo_2020 | 98 | Brazil | 107 | HD | 48.91+/-46.81 months | 54.30+/-14.85 | 67.3 |
| Gopinathan_2020 | 99 | India | 39 | HD | Not reported | 78.03+/-3.90 | 79.5 |
| Goto_2019b | 70 | Netherlands | 187 | HD; PD | Initiation | 75+/-7 | 67 |
| Goto_2019 | 71 | Netherlands | 285 | HD; PD; Conservative | Initiation | 78+/-7 | 64 |
| Hasegawa_2020 | 100 | Japan | 2404 | HD | 3.8 (0.7-9.4) years | 61.6+/-12.4 | 62.9 |
| Jafari_2020b | 105 | Canada | 100 | HD | 35.5 (13.75-71.75) months | 63+/-15 | 58 |
| Jafari_2021 | 106 | Canada | 109 | HD | 34.0 (13.0-67.0) months | 63.3+/-14.2 | 61.5 |
| Johansen_2007 | 81 | USA | 2275 | HD; PD | Initiation | 58.2+/-15.5 | 53.4 |
| Johansen_2014b | 107 | USA | 638 | HD | 3.2 (0.1-36.6) years | 57.1+/-14.3 | 58.6 |
| Johansen_2015 | 108 | USA | 68 | HD | 3.7 (1.6-6.3) years | 59+/-14 | 59 |
| Johansen_2016 | 109 | USA | 762 | HD | 2.7 (1.2, 5.4) years | 57.1+/-14.2 | 59.3 |
| Johansen_2019 | 110 | USA | 771 | HD | Not reported | 57.2+/-14.3 | 59.2 |
| Kang_2017 | 78 | Korea | 1611 | HD; PD | Not reported |  |  |
| Kimura_2021 | 112 | USA | 337 | HD | Not reported | 56+/-13 | 55 |
| Kutner_2014c | 115 | USA | 742 | HD | Median 3 years | 57.2 +/-14.1 | 59.4 |
| Li_2021 | 116 | China | 150 | HD | Not reported | 69 (IQR 64-75) | 48.7 |
| McAdams-DeMarco_2013 | 121 | USA | 95 | HD | Median 3.7 years | 60.5 +/-12.6 | 53.7 |
| McAdams-DeMarco_2015 | 122 | USA | 324 | HD | Not reported | 54.8 +/-13.3 | 43.5 |
| McAdamsDeMarco_2013c | 9 | USA | 146 | HD | Not reported | 60.6 +/- 13.6 | 53.4 |
| Miyazaki_2021 | 123 | Japan | 20 | HD | 91.7+/-80.1 months | 76.5 (65-88) | 88 |
| Nakazato_2020 | 125 | Japan | 109 | HD | 11.3+/-8.4 years | 65.6+/-12.3 | 67.9 |
| Noori_2018 | 126 | Canada | 151 | HD | 6.2(0.3-3.0) years | 64+/-14 | 56 |
| Okuyama_2018 | 127 | Japan | 362 | HD | 11.1+/-9.0 years | 71.1+/-9.6 | 59.7 |
| Painter_2013b | 129 | USA | 188 | HD | 34.2+/-38.7 months | 55.7+/-15.3 | 43.6 |
| Salter_2015 | 25 | USA | 146 | HD | 3.6 (1.4-6.4) years | 61(53-70) | 53.4 |
| Sy_2019 | 133 | USA | 771 | HD | Not reported | 57.2+/-14.2 | 59.4 |
| Takeuchi_2018 | 134 | Japan | 388 | HD | 8.7+/-7.6 years | 67.2+/-11.9 | 62.4 |
| Usui_2021 | 135 | Japan | 158 | HD | 5.5 (2.5-10.2) years | 74.1+/-6.8 | 71.5 |
| Yabuuchi_2020 | 137 | Japan | 37 | HD | 8.9 years | 67.2 | 59.4 |
| Yadla_2017 | 138 | Saudi Arabia | 205 | HD | 2.5+/-1.3 years | 44.95+/-13.27 | 69.2 |
| Zanotto_2020 | 143 | UK | 69 | HD | 713+/-714 days | 61.7+/-13.3 | 55.1 |
| Zanotto_2021 | 144 | UK | 76 | HD | Not reported | 61.1 (SD14) | 53.9 |
| van_Loon_2017 | 136 | Netherlands | 123 | HD | Initiation | 76+/-7 | 64 |
| van_Loon_2019b | 83 | Netherlands | 192 | HD; PD | Initiation | 75+/-7 | 67 |

Supplementary Table 5: Studies examining patient populations undergoing haemodialysis using Fried assessment of frailty.

PD

| Reference name | Reference number | Country | Sample size | Nephrology population | Dialysis vintage | Patient age  Mears Years +/- SD  Median years (range) | Patient gender (% male) |
| --- | --- | --- | --- | --- | --- | --- | --- |
| Bao_2012 | 6 | USA | 1576 | HD; PD | Initiation | 59.6+/-14.2 | 55.5 |
| Drost_2016 | 80 | Netherlands | 95 | HD; PD; GFR<30 | Not reported | 65.2+/-12.0 |  |
| Farragher_2019 | 151 | Canada | 121 | PD | Initiation | 69.2+/-10.2 | 67 |
| Goto_2019 | 71 | Netherlands | 285 | HD; PD; Conservative | Initiation | 78+/-7 | 64 |
| Goto_2019b | 70 | Netherlands | 187 | HD; PD | Initiation | 75+/-7 | 67 |
| Johansen_2007 | 81 | USA | 2275 | HD; PD | Initiation | 58.2+/-15.5 | 53.4 |
| van_Loon_2019b | 83 | Netherlands | 192 | HD; PD | Initiation | 75+/-7 | 67 |

Supplementary Table 6: Studies examining patient populations undergoing peritoneal dialysis using Fried assessment of frailty.

Transplant candidates

| Reference name | Reference number | Country | Sample size | Nephrology population | Dialysis vintage | Patient age  Mears Years +/- SD  Median years (range) | Patient gender (% male) |
| --- | --- | --- | --- | --- | --- | --- | --- |
| Haugen_2019 | 197 | USA | 7078 | transplant_candidate | Not reported | 54+/-13 | 60 |
| Haugen_2020 | 156 | USA | 6379 | Transplant; transplant_candidate | Not reported | Not reported |  |
| Haugen_2021 | 157 | USA | 1154 | Transplant; transplant_candidate | Not reported | 54+/-13 | 65.9 |
| Lorenz_2019b | 160 | USA | 272 | transplant_candidate | 22.4+/-27.7 months | 61.8 +/- 9.3 | 62.1 |
| McAdams-DeMarco_2018 | 164 | USA | 1975 | transplant_candidate | Not reported | 53.7 +/-13.5 | 59.5 |
| McAdamsDeMarco_2015b | 162 | USA | 537 | transplant_candidate | Not reported | 53.0 +/-14.0 | 60 |
| Novais_2021 | 165 | France | 156 | transplant_candidate | Not reported | 74.2+/-3.5 | 69.2 |
| Perez_Fernandez_2019 | 166 | USA | 2086 | transplant_candidate | Not reported | 53.8+/-13.5 | 59.9 |
| Shrestha_2019 | 169 | USA | 1003 | transplant_candidate | Not reported | 55 +/-13 | 60 |

Supplementary Table 7: Studies examining patient populations assessed for kidney transplant candidacy using Fried assessment of frailty.

Transplant recipients

| Reference name | Reference number | Country | Sample size | Nephrology population | Dialysis vintage | Patient age  Mears Years +/- SD  Median years (range) | Patient gender (% male) |
| --- | --- | --- | --- | --- | --- | --- | --- |
| Garonzik-Wang_2012.pdf | 174 | USA | 183 | Transplant | 2.5+/-3.9 years | 53.4+/-14.0 | 63.7 |
| Haugen_2021.pdf | 157 | USA | 1154 | Transplant; transplant_candidate | Not reported | 54+/-13 | 65.9 |
| Konel_2018.pdf | 175 | USA | 773 | Transplant | Not reported | 54+/-14 | 62.2 |
| McAdams-DeMarco_2015f.pdf | 179 | USA | 525 | Transplant | Not reported | 53.0 +/-14.0 | 60.2 |
| McAdams-DeMarco_2017.pdf | 180 | USA | 663 | Transplant | Not reported | 53.0 +/-13.9 | 62 |
| McAdamsDeMarco_2013.pdf | 177 | USA | 383 | Transplant | Not reported | 53.5+/-13.9 | 61.1 |
| McAdamsDeMarco_2015c.pdf | 178 | USA | 349 | Transplant | 2.1 years (IQR 0.4-3.9) | 53.3+/- 14.2 | 61.9 |
| Nastasi_2018.pdf | 182 | USA | 719 | Transplant | Not reported | 51.6+/-14.2 | 62.3 |
| dos_Santos_Mantovani_2020.pdf | 173 | Brazil | 87 | Transplant | 2.5+/-3.9 years | 53.4+/-14.0 | 63.7 |

Supplementary Table 8: Studies examining patient populations with kidney transplant using Fried assessment of frailty
